# Farnesol, a Dietary Sesquiterpene, Attenuates Rotenone-Induced Dopaminergic Neurodegeneration by Inhibiting Oxidative Stress, Inflammation, and Apoptosis via Mediation of Cell Signaling Pathways in Rats

**DOI:** 10.3390/ijms27020811

**Published:** 2026-01-14

**Authors:** Lujain Bader Eddin, Seenipandi Arunachalam, Sheikh Azimullah, Mohamed Fizur Nagoor Meeran, Mouza Ali Hasan AlQaishi Alshehhi, Amar Mahgoub, Rami Beiram, Shreesh Ojha

**Affiliations:** 1 Department of Pharmacology and Therapeutics, College of Medicine and Health Sciences, United Arab Emirates University, Al Ain P.O. Box 15551, United Arab Emirates; 201970113@uaeu.ac.ae (L.B.E.); 201570353@uaeu.ac.ae (M.A.H.A.A.);; 2 Department of Pharmaceutical Biosciences, Drug Safety and Toxicology, Uppsala Biomedicines Centrum BMC, Husarg, 75124 Uppsala, Sweden; 3 Zayed Bin Sultan Center for Health Sciences, United Arab Emirates University, Al Ain P.O. Box 15551, United Arab Emirates

**Keywords:** Parkinson’s disease, rotenone, oxidative stress and inflammation, apoptosis, sesquiterpene, farnesol

## Abstract

Parkinson’s disease is a neurodegenerative disorder that affects the elderly population worldwide. Rotenone (ROT) is an environmental toxin that impairs mitochondrial dynamics by inhibiting respiratory chain complex I and thus inducing oxidative stress. Farnesol (FSL) is a dietary sesquiterpene with antioxidant and anti-inflammatory properties reported in various in vivo models. To evaluate the efficacy of FSL in the management of PD, Wistar rats were injected with ROT (2.5 mg/kg, i.p) and pretreated with FSL. Immunohistochemical staining measured tyrosine hydroxylase-positive cells in the substantia nigra and striatum. Western blotting was employed to determine protein expression of inflammatory, apoptotic, and autophagic markers. Our results indicate that FSL significantly protected against ROT-induced inflammation by suppressing microglial and astrocytic activation through the downregulation of Toll-Like receptor 4 (TLR4), nuclear factor kappa-light-chain-enhancer of activated B cells (NF-κB), inhibitor of kappa B (IkB), inducible nitric oxide synthase (iNOS), cyclooxygenase (COX), matrix metalloproteinase-9 (MMP-9) expression. FSL has also demonstrated an antioxidant effect by enhancing the activity of superoxide dismutase and catalase while reducing the level of Malondialdehyde and nitric oxide. Moreover, it restored homeostasis in ROT-induced imbalance between pro- and anti-apoptotic proteins. Impaired autophagy observed in ROT-injected rats was corrected by FSL treatment, which upregulated phosphorylated mammalian target of rapamycin (p-mTOR) expression and downregulated P62, an autophagosome marker. The protective effect of FSL was further supported by preserving the brain-derived neurotrophic factor (BDNF) and tyrosine hydroxylase in the brain. These findings demonstrate the neuroprotective ability of FSL and its potential to be developed as a pharmaceutical or nutraceutical agent for the prevention and treatment of PD by mitigating neuropathological changes observed in dopaminergic neurodegeneration.

## 1. Introduction

Parkinson’s disease (PD) is a chronic neurodegenerative disorder that affects motor coordination, muscle movements, and cognitive abilities. A variety of pathogenic factors play a role in the development of PD, including oxidative stress, neuroinflammation, mitochondrial impairment, apoptotic cell death, and dysregulated autophagy [1,2]. Persistent overproduction of neurotoxic factors and free radicals triggers chronic neuroinflammation, which promotes dopaminergic neuron degeneration and worsens PD symptoms [3]. Activation of microglial and astrocytes has been well-documented in PD, leading to the release of proinflammatory cytokines/mediators, interleukin-6 (IL-6), interleukin-1β (IL-1β), tumor necrosis factor-alpha (TNF-α), and oxygen free radicals [4]. Microgliosis and astrogliosis also induce the expression of COX-2 and inducible iNOS, key enzymes involved in neurotoxicity and oxidative stress [5,6,7,8].

Mitochondrial dysfunction through the inhibition of respiratory chain complexes results in oxidative and nitrative stress, which contributes to pathological accumulation of α-synuclein [9]. In turn, excessive α-synuclein aggravates free radical generation and impairs mitochondrial respiration, establishing a vicious cycle that accelerates PD pathology. Emerging evidence also highlights the role of TLRs and neuroinflammation in PD pathogenesis. TLRs are expressed on immune, neuronal, and glial cells and activated by pathogen-associated molecular patterns, initiating a downstream signaling cascade involving NF-κB activation, promoting the release of proinflammatory cytokines such as IL-1β, IL-6, and TNF-α [10].

Another disrupted pathway in PD involves neuronal homeostasis regulated by the mTOR signaling pathway [11]. This pathway modulates phosphorylation of downstream effectors, including p70s6 kinase, eukaryotic initiation factor 4E binding protein 1 (4E-BP1), B cell leukemia/lymphoma 2 (Bcl2), and proapoptotic protein (Bax), which collectively regulate mitochondrial membrane permeability and the intrinsic apoptotic pathway [12].

Rotenone (ROT), a naturally occurring pesticide, has been recognized as a potent mitochondrial toxin since the 1960s [13]. It is a well-established mitochondrial complex I inhibitor that closely mimics key pathological features of PD. By interfering with the electron transport chain, ROT causes impaired ATP synthesis and excessive production of reactive oxygen and nitrogen species. ROT exposure also induces α-synuclein aggregation, microglial activation, and dopaminergic neuronal loss, recapitulating both biochemical and behavioral abnormalities observed in PD [14]. Furthermore, it is highly lipophilic, allowing it to easily cross the blood–brain barrier, which enhances its neurotoxic potency and makes it a widely used agent for modeling PD-like neurodegeneration [15].

In recent years, attention has turned toward natural bioactive compounds with antioxidant and anti-inflammatory properties as potential neuroprotective agents. Farnesol (FSL), a plant-derived sesquiterpene alcohol found in essential oils of several spices, has emerged as a promising candidate due to its diverse pharmacological activities. FSL exerts potent free radical scavenging, attenuates lipid peroxidation, and upregulates endogenous antioxidant defenses such as SOD and glutathione (GSH). Moreover, FSL has been reported to suppress proinflammatory cytokine production, downregulate NF-κB signaling, and modulate the apoptotic pathway, thereby preserving neuronal integrity [16,17]. Additionally, it has been shown to improve cognitive behavior, inhibit microglial activation in the hippocampus and neuroinflammation, and mitigate oxidative stress in an animal model of Alzheimer’s disease [18]. FSL is a geometric isomer of Nerolidol, commonly found in the essential oils of many plants, and Nerolidol has also shown effectiveness in the experimental models of ROT-induced dopaminergic neurodegeneration [19].

Given the protective effects of FSL against the main mechanistic pathways implicated in PD and its isomeric characteristics with Nerolidol, FSL’s multitargeted protective mechanisms may provide significant therapeutic benefit. Therefore, the present study aimed to investigate the neuroprotective potential of FSL against ROT-induced dopaminergic neurodegeneration and to elucidate the underlying pharmacological mechanisms contributing to its protective effects.

## 2. Results

### 2.1. Effect of FSL on Oxidative Stress Markers

**Figure 1:**
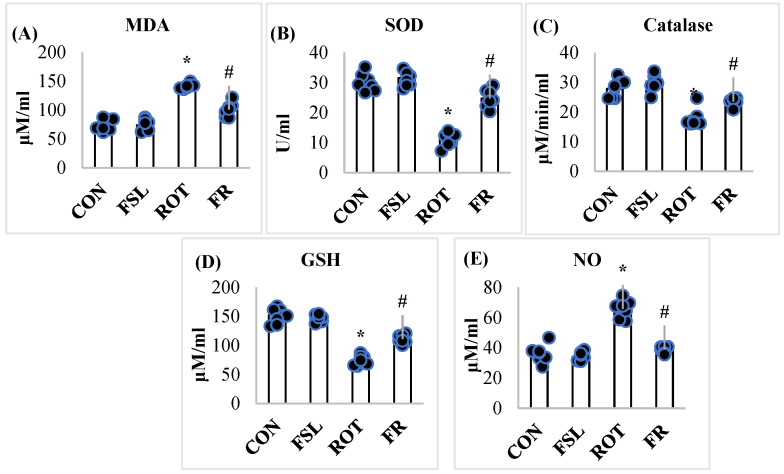
Effect of FSL on the levels/concentrations of MDA, SOD, catalase, GSH, and NO in the midbrain of ROT-injected rats (**A**–**E**). Data presented as mean ± SEM (*n* = 8); * *p* < 0.05 CON vs. ROT; # *p* < 0.05 ROT vs. FSL + ROT (One-way ANOVA followed by DMRT). CON: normal control; FSL: farnesol control; ROT: rotenone; FSL + ROT: farnesol + rotenone.

A compromised antioxidant defense is marked by an imbalance of ROS and a reduction in the corresponding antioxidant countering the free radicals. ROT injections increased the level of MDA and NO in the midbrain compared to the CON group. However, the co-administration of FSL to ROT-injected rats showed a significant decrease (*p* < 0.05) in the levels of MDA and NO in the midbrain, compared to the ROT group. On the other hand, ROT-injected rats showed a significant reduction in the concentration/activities of GSH, SOD, and catalase when compared to the CON group, whereas FSL treatment significantly (*p* < 0.05) increased the concentration/activities of GSH, SOD, and catalase (Figure 1A–E).

### 2.2. Effect of FSL on the Levels/Expressions of Proinflammatory Cytokines in the Midbrain and Striatum

**Figure 2:**
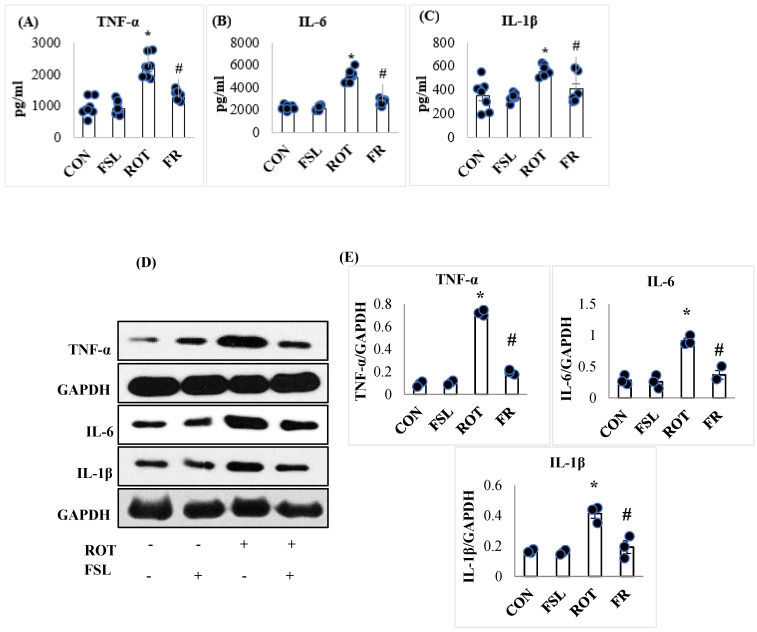
Effect of FSL on the levels of proinflammatory cytokines (TNF-α, IL-6, and IL-1β) in the midbrain of rats (**A**–**C**). (**D**,**E**) Immunoblotting and densitometric analysis of the protein expression of proinflammatory cytokines in the striatum. Immunoblotting analysis was performed in triplicate (*n* = 3). Data presented as mean ± SEM (*n* = 8); * *p* < 0.05 CON vs. ROT; # *p* < 0.05 ROT vs. FSL + ROT (One-way ANOVA followed by DMRT). CON: normal control; FSL: farnesol control; ROT: rotenone; FSL + ROT: farnesol + rotenone.

The level of the proinflammatory cytokines (TNF-α, IL-1β, and IL-6) was significantly (*p* < 0.05) increased following ROT injections in the midbrain of rats when compared to the CON group. However, when compared to the ROT group, the level of proinflammatory cytokines was significantly reduced in rats pre-treated with FSL following ROT injections (Figure 2A–C). Consistent with the levels, a considerable rise in the expression of TNF-α, IL-1β, and IL-6 was observed in the ROT group compared to the CON group, as demonstrated by Western blot analysis. On the other hand, pre-treatment with FSL prevented ROT-induced rise in the proinflammatory markers in the striatum compared to the ROT group (Figure 2D,E).

### 2.3. Effect of FSL on the Expressions/Levels of Inflammatory Mediators, Including (TLR4, NF-κB, IκB, iNOS, COX-2, and MMP-9)

**Figure 3:**
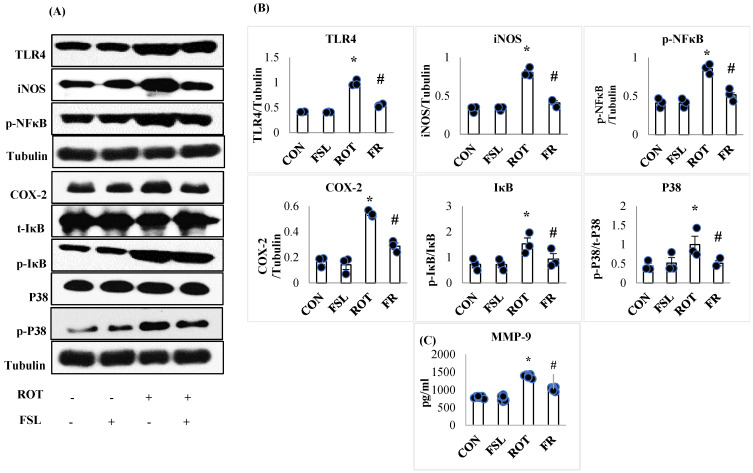
Immunoblotting analysis and quantification of TLR4, iNOS, COX-2, NF-κB, p-NF-κB, t-IκB, p-IκB, P38, and p-P38 in the striatal tissues of rats (**A**,**B**). Immunoblotting was performed in triplicate (*n* = 3). (**C**) Effect of FSL on the expressions of MMP-9 in the midbrain (*n* = 8) and the results are shown as the mean ± SEM (*n* = 8); * *p* < 0.05 CON vs. ROT; # *p* < 0.05 ROT vs. FSL + ROT (One-way ANOVA followed by DMRT). CON: normal control; FSL: farnesol control; ROT: rotenone; FSL + ROT: farnesol + rotenone.

The onset and progression of inflammation is initiated following activation of the transmembrane receptors, TLR4, and further augmented by the induced intracellular signaling, which transduces the transcription activity of various transcription factors, increasing the expression of proinflammatory mediators. To study the effect of FSL on ROT-induced neuroinflammation, the protein expression of TLR4, NF-κB, IκB, iNOS, p-P38, and COX-2 was carried out in the striatum of PD rats. ROT-injected rats displayed a significant (*p* < 0.05) rise in the expression of all these proteins, instituting inflammation when compared to the CON group. However, the rise in the expression was reversed by FSL treatment of ROT-injected rats (Figure 3A,B). As demonstrated in Figure 3C, MMP-9 expression was disrupted following ROT injections. Rats injected with ROT displayed an increase in the expression level of MMP-9 that was reinstated by the administration of FSL. The expression level of inflammatory mediators in CON and FSL-treated rats was observed to be unaltered, which depicts a normal expression level.

### 2.4. Effect of FSL on the Activation of Microglia and Astrocytes in the Striatum

**Figure 4:**
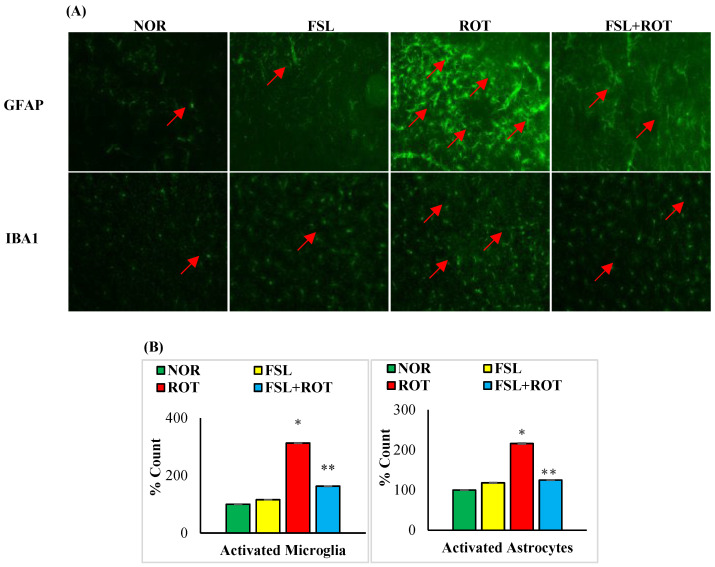
The activation of GFAP and Iba-1 in the striatum was explored by immunofluorescence staining of the striatum (**A**). A remarkable expression of activated astrocytes (GFAP-positive) and microglia (Iba-1-positive) was indicated in the fluorescent images taken from ROT-injected rats when compared to the control rats (scale bar = 200 µm) (Red arrows indicate GFAP and IBA1 immunopositive cells). The quantitative analysis of the number of activated astrocytes and microglia is shown (**B**). Experimental groups consist of seven rats each, and the values are presented as mean ± SEM (*n* = 7); * *p* < 0.05 NOR vs. ROT; ** *p* < 0.05 ROT vs. FSL + ROT (One-way ANOVA followed by DMRT). NOR: normal control; FSL: farnesol control; ROT: rotenone; FSL + ROT: farnesol + rotenone.

Microglia and astrocytes are the main immune cells in the brain that are considered one of the major sources of inflammatory mediators. The immunofluorescence staining of GFAP and Iba-1 showed a significant (*p* < 0.05) increase in the number of activated GFAP-positive astrocytes (Figure 4A) and Iba-1-positive microglia cells (Figure 4B) in ROT-injected rats when compared to the normal control group. The co-administration of FSL to ROT-injected rats showed a significant (*p* < 0.05) reduction in the activation of both microglia and astrocyte cells in the striatum of rats when compared to the ROT group. There was no notable expression of activated microglia and astrocyte cells in normal control rats and rats treated with FSL only.

### 2.5. Effect of FSL on Dopaminergic Neurodegeneration in Striatum and Substantia Nigra

**Figure 5:**
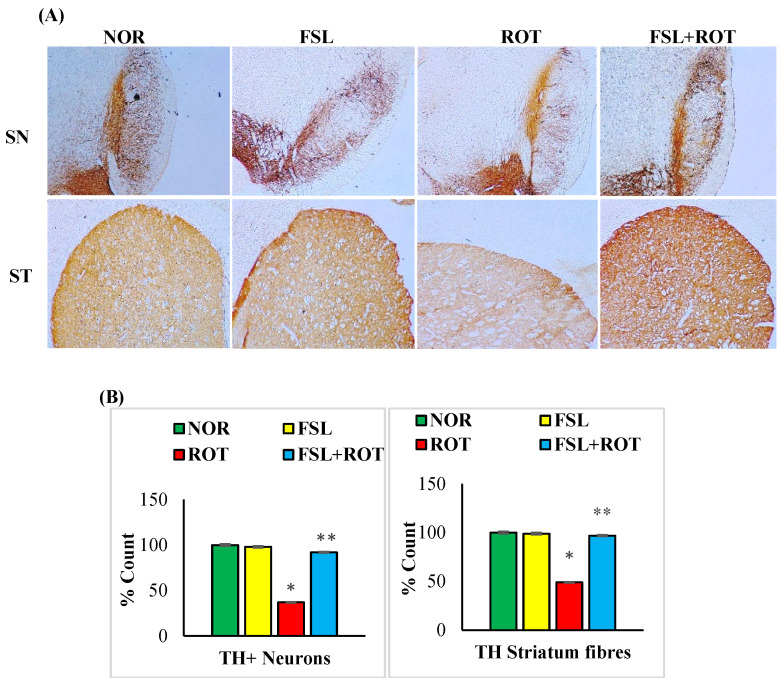
TH-ir neurons and TH-ir fibers in the substantia nigra (SN) and striatum are presented, respectively (**A**) (Scale bar is 100 µm). Quantification of the number of TH-ir neurons in SNc and the density of TH-ir fibers is also shown (**B**). Groups consist of seven rats each, and the values are presented as mean ± SEM (*n* = 7); * *p* < 0.05 NOR vs. ROT; ** *p* < 0.05 ROT vs. FSL + ROT (One-way ANOVA followed by DMRT). NOR: normal control; FSL: farnesol control; ROT: rotenone; FSL + ROT: farnesol + rotenone.

Selective degeneration of the dopamine-producing neurons is the central manifestation of PD that is ascribed to the persistent oxidative stress and inflammation. ROT injections caused a significant (*p* < 0.05) depletion in the number of TH-ir neurons in the SN (Figure 5A) and the density of TH striatal fibers (Figure 5B) in comparison with the CON group. FSL treatment of ROT-injected rats showed salvage of the dopaminergic neurons in SN and their extensional fibers in the striatum, as evident by the increased immunoreactivity to the TH-positive cells in comparison with the ROT-only group. The rats in the CON group and FSL-only treatment showed an increased content of both TH-ir neurons and fibers in the SN and striatum, respectively.

### 2.6. Effect of FSL on the Expression of Brain-Derived Neurotrophic Factor (BDNF), Tyrosine Hydroxylase, and α-Synuclein in Striatum

**Figure 6:**
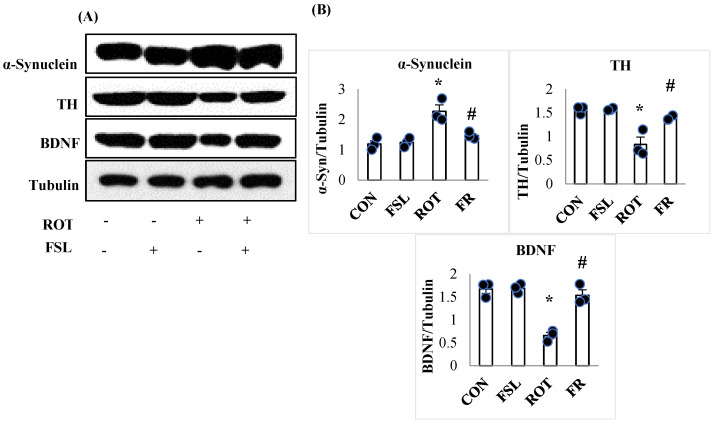
Representative images of Western immunoblots and densitometric analysis for TH, BDNF, and α-synuclein expressions in the striatal tissues (**A**,**B**). Immunoblotting analysis was performed in triplicate (*n* = 3). * *p* < 0.05 CON vs. ROT; # *p* < 0.05 ROT vs. FSL + ROT (One-way ANOVA followed by DMRT). CON: normal control; FSL: farnesol control; ROT: rotenone; FSL + ROT: farnesol + rotenone.

Aberrantly aggregated α-Synuclein and the insufficient reservoir of BDNF and TH are considered one of the key mediators of dopaminergic neurodegeneration in manifesting PD. ROT injections were associated with a significant (*p* < 0.05) fall in the expression of BDNF and TH enzyme in the striatum when compared to the CON group. However, FSL treatment of ROT-injected rats preserved the expression of both BDNF and TH when compared to their expression in the ROT group. In contrast, there was a significant (*p* < 0.05) rise in the expression of α-Synuclein protein in ROT-injected rats in comparison with the CON group, which depicts accumulation of α-Synuclein. Rats injected with ROT upon treatment with FSL showed a significant downregulation of α-Synuclein expression, displayed by reduced accumulation when compared to the ROT group (Figure 6).

### 2.7. Effect of FSL on the Expression of Mitochondrial Complex I

**Figure 7:**
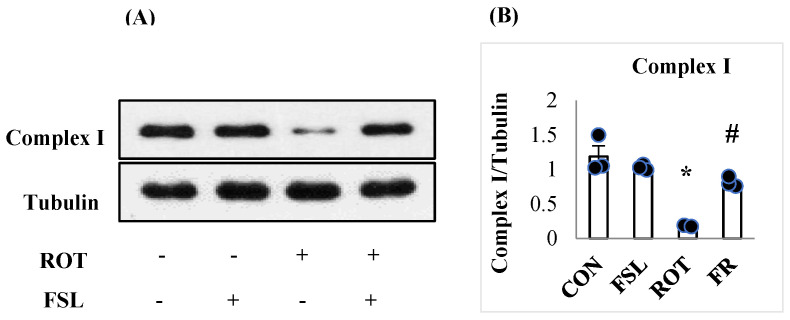
Western immunoblotting and densitometric analysis on the protein expression of mitochondrial complex I in the striatal tissues of rats (**A**,**B**). Immunoblotting analysis was performed in triplicate (*n* = 3). * *p* < 0.05 CON vs. ROT; # *p* < 0.05 ROT vs. FSL + ROT (One-way ANOVA followed by DMRT). CON: normal control; FSL: farnesol control; ROT: rotenone; FSL + ROT: farnesol + rotenone.

The effect of ROT on mitochondrial complex I was assessed by measuring its expression. A significant (*p* < 0.05) reduction in complex I in ROT-injected rats compared to CON rats showed the ROT-induced alteration in mitochondrial activities. In contrast, in ROT-injected rats treated with FSL, a significant (*p* < 0.05) restoration of complex I in comparison with the ROT group was observed (Figure 7). Rats of the normal control group (CON) and FSL-treated group did not show a change in the expression of mitochondrial complex I.

### 2.8. Effect of FSL on the Expression of Apoptotic Markers

**Figure 8:**
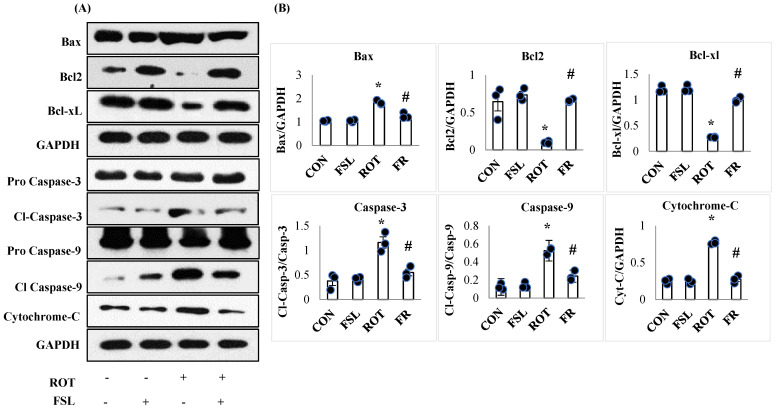
Immunoblotting and densitometric analysis of apoptotic marker proteins in the striatum (**A**,**B**). Immunoblotting was performed in triplicate, and the results are presented as the mean ± SEM (*n* = 3). * *p* < 0.05 CON vs. ROT; # *p* < 0.05 ROT vs. FSL + ROT (One-way ANOVA followed by DMRT). CON: normal control; FSL: farnesol control; ROT: rotenone; FSL + ROT: farnesol + rotenone.

Inefficient antioxidant power following overt generation of free radicals and persistent neuroinflammatory insult is the primary reason for apoptotic cell death, leading to neuronal loss. ROT-induced neuronal injury is associated with an increased expression of apoptotic proteins. The expression of pro-apoptotic proteins, including BAX, caspase-3, caspase-9, and cytochrome-C, was observed to be upregulated (*p* < 0.05) in ROT-injected rats when compared to the CON group. However, ROT-injected rats pretreated with FSL showed mitigation of apoptosis, evident by reduced expression of the pro-apoptotic proteins when compared to the ROT group (Figure 8). On the other hand, the protein levels of antiapoptotic protein, Bcl2, and Bcl-xL were significantly (*p* < 0.05) downregulated in rats administered ROT injections when compared to the CON group. FSL treatment of ROT-injected rats showed a significant (*p* < 0.05) rise in the expression of these anti-apoptotic proteins in comparison with the ROT group (Figure 8).

### 2.9. Effect of FSL on Autophagic Markers

**Figure 9:**
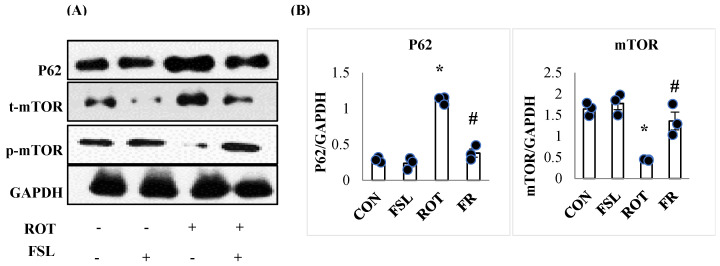
Immunoblotting and densitometric analysis of P62, m-TOR, and p-m-TOR in the striatal tissues (**A**,**B**). Immunoblotting was performed in triplicate (*n* = 3). * *p* < 0.05 CON vs. ROT; # *p* < 0.05 ROT vs. FSL + ROT (One-way ANOVA followed by DMRT). CON: normal control; FSL: farnesol control; ROT: rotenone; FSL + ROT: farnesol + rotenone.

PD pathogenesis is also attributed to impaired autophagy associated with impaired ubiquitination of damaged organelles. Rats injected with ROT exhibited a remarkable (*p* < 0.05) decline in autophagy regulator p-mTOR expressed as a ratio of p-mTOR/mTOR and increased ubiquitin molecule P62 (Figure 9). FSL treatment of ROT-injected rats showed improved expression of autophagy mediator, following an increased ratio of p-mTOR/mTOR and a downregulated ubiquitin molecule autophagosome marker, P62 (Figure 9).

## 3. Discussion

Parkinson’s disease (PD) is one of the common neurodegenerative disorders worldwide. It is characterized by the degeneration of the dopaminergic neurons and the accumulation of α-synuclein in the SN and other anatomical regions of the brain. Current treatment approaches can alleviate associated symptoms, but offer neither a permanent cure nor strategies to halt disease progression [20,21]. Therefore, given the multifactorial nature of PD pathology, novel therapies and agents with multiple pharmacological properties are considered vital in offering significant benefit. In the present study, we used the ROT-based PD model, which mimics major features of PD [14]. This model is well-established and popularly utilized for evaluating the neuroprotective ability of agents and underlying pharmacological and molecular mechanisms implicated in the pathogenesis of PD. The neuropathology of PD is manifested by degeneration of dopaminergic neurons in the SN and subsequent denervation of the striatum [22]. Our results demonstrated that ROT injections elicit a significant decline in the number of dopaminergic neurons in the SN and dopaminergic fibers in the striatum, evident by the decreased staining intensity in histology. However, FSL treatment protected against ROT-induced loss of dopaminergic neurons, as indicated by enhanced neuronal reactivity in the striatum and SN.

Studies have shown that neuroinflammation plays a major role in PD pathogenesis, mediated by activated immune cells in the brain, including microglia and astrocytes. While microglia normally exert neuroprotective functions in the brain, their excessive activation triggers a detrimental cycle of neuroinflammation that contributes to PD [4]. ROT in the present study is observed to induce the activation of microglia cells in the brain through the activation of the NF-κB pathway and the translocation of phosphorylated p65 subunit of NF-κB to the nucleus. The present study results are consistent with numerous previously published studies wherein ROT has been demonstrated to provoke a neuroinflammatory cascade [23,24]. ROT injections increased the expression of phosphorylated NF-κB, as well as the number of activated microglia cells observed through fluorescence immunohistochemistry. FSL administration showed a marked attenuation in ROT-induced inflammation by decreasing the activation of microglia and inhibiting the NF-κB pathway. Moreover, TLR is expressed on immune cells and therefore participates in the onset and progression of neuroinflammation. α- Synuclein acts as a damage-associated molecular pattern that activates TLRs, priming microglia cells, cytokines, and neuroinflammation initiation [25]. It has also been reported that TLR expression is upregulated in the SN of PD patients [26]. ROT is found to increase the expression of TLR4 [27], which is also observed in the present study. In addition, matrix metalloproteinases such as MMP-2,9 are also implicated in neuroinflammation progression through activating inflammatory pathways and stimulating the release of cytokines, chemokines, and adhesion molecules. These proinflammatory molecules have been implicated in the impairment of the blood–brain barrier through the disruption of tight junctions, which connect adjacent endothelial cells, disrupting them and leading to basement membrane extravasation [28]. ROT injection is demonstrated to induce an inflammatory cascade through enhancing MMP-9 activity with a concurrent increased release of inflammatory mediators (iNOS, COX-2) and cytokines (IL-1β, IL-18, and TNF-α). FSL treatment conferred neuroprotection through suppressing TLR-4 activation, inhibiting MMP-9, with a subsequent reduction in the release of iNOS, COX2, IL-1β, IL-18, and TNF-α. Additionally, P38 is part of the mitogen-activated protein kinase (MAPK) intracellular signaling pathway that mediates the activation of intrinsic inflammatory pathways within the cell. The inhibition of P38 signaling is protective for preserving dopaminergic neurons through blocking the augmentation of inflammation [29]. Our results indicated that the FSL inhibited ROT-induced activation of P38 signaling.

Complex I plays a critical role in establishing the proton gradient across mitochondrial complexes. Its inhibition disrupts electron transport, leading to excessive ROS generation, a principal mechanism underlying ROT neurotoxicity. ROT is a selective complex I inhibitor [30]. The present findings indicate an elevated oxidative stress response in the striatum of ROT-injected rats, evidenced by increased lipid peroxidation level. These results align with previous reports [31,32] demonstrating an impaired antioxidant defense following ROT administration. The antioxidant defense system, consisting of both enzymatic and non-enzymatic components such as SOD, catalase, and GSH, represents the primary line of defense against free radical-induced damage [33]. The diminished antioxidant defense, characterized by reduced levels of GSH, catalase, and SOD, is recognized as a contributing factor in PD pathogenesis [34]. The results of our study showed a potential antioxidant effect of FSL in maintaining antioxidant defense against ROT-induced free radicals. In previous studies, the antioxidant and membrane-stabilizing effects of FSL have been reported in organ injuries induced by xenobiotics in experimental models mimicking diseased conditions [35,36]. This illustrates the free radical scavenging activity of FSL against the generated ROS induced by ROT.

Apoptosis is a regulated process of cell death and is also implicated in the causation of numerous diseases and disease models in response to cellular injury. Excessive cellular levels of ROS damage cellular proteins, membranes, and organelles, predisposing to apoptotic death [37]. ROT induces apoptosis due to its chronic inhibition of respiratory chain complex I and the subsequent generation of mitochondrial ROS, release of cytochrome c, and caspase-3 activation [38]. In this context, FSL treatment of ROT-injected rats mitigated dopaminergic neuronal loss by restoring the balance between pro- and anti-apoptotic proteins. ROT administration activated mitochondrial-dependent apoptotic pathway, leading to increased expression of Bax, caspase 3, 9, and cytochrome C in the striatum. Notably, FSL treatment favorably modulated this response by suppressing proinflammatory cytokine expression and concomitantly upregulating Bcl-2 and Bcl-xL.

Autophagy is a critical cellular process responsible for eliminating misfolded and aggregated proteins formed in various synucleinopathies, thereby preserving neuronal homeostasis. Impaired autophagy disrupts cellular equilibrium and decreases neuronal survival, contributing significantly to PD pathogenesis. Studies reported that autophagosomes are increasingly accumulated in ROT-injected rodents, indicative of an accelerated autophagic response [39,40]. It is also reported that the build-up of autophagic vacuoles, if not regularly eliminated, is toxic to the dopaminergic neurons [41]. Rodent studies have demonstrated that ROT administration increases the formation of autophagic vacuoles, as indicated by elevated LC3 expression. Concurrently, p62 levels were also elevated, suggesting impaired lysosomal degradation and the subsequent accumulation of autophagic vacuoles [42]. This dysregulated autophagy promotes apoptosis through the lysosomal release of proapoptotic proteins [43]. mTOR serves as a central regulator of cellular survival, proliferation, and metabolism. Under oxidative stress, ROS-mediated protein damage enhances the need for detoxification processes, including autophagy, to eliminate defective components and maintain cellular integrity [44]. However, impaired autophagy contributes to a vicious cycle of protein aggregation and neuronal degeneration, as widely documented in PD [39]. In PD models, mTOR signaling is often suppressed following exposure to neurotoxins such as ROT [45,46]. Inhibition of mTOR enhances autophagy in an attempt to clear toxic molecules; however, this compensatory response can become maladaptive, leading to increased autophagic activity, delayed clearance of toxic particles, and eventual neuronal loss [47]. The present study showed that ROT inhibited the mTOR pathway, whereas FSL treatment restored normal mTOR signaling. Moreover, P62 (also known as Sequestosome-1), a key autophagy adaptor protein that directs ubiquitinated substrates for degradation, is accumulated in response to ROT-induced autophagic disruption and mTOR downregulation [48]. FSL treatment of ROT-challenged rats markedly decreased P62 expression, indicating an improvement in autophagic flux and cellular homeostasis.

α-synuclein is an intrinsically disordered monomeric protein predominantly expressed in the brain, though it can also exist in tetrameric form. The interaction between the positively charged N-terminus and the negatively charged C-terminus exposes aggregation-prone components of the protein, facilitating protein unfolding. An imbalanced tetramer-to-monomer ratio favors the formation of β-sheet oligomers that accumulate as Lewy bodies within neuronal axons in PD [49]. α-synuclein plays a key role in regulating synaptic vesicle trafficking and exocytosis; however, its overexpression disrupts vesicular homeostasis. The neuropathological alterations induced by ROT are characterized by progressive aggregation of misfolded α-synuclein preceding neuronal loss [50]. The present study showed a marked expression of α-synuclein in ROT-injected rats, whereas FSL treatment attenuated ROT-induced α-synuclein accumulation.

Moreover, BDNF is essential for maintaining neuronal integrity by promoting neurogenesis, synaptogenesis, and synaptic plasticity. Reduced BDNF level has been reported in PD, suggesting impaired trophic support [51]. ROT administration has been shown to alter BDNF expression, thereby mimicking the early pathological stages of PD [52,53]. Consistent with this, our findings revealed a significant decline in BDNF level following ROT exposure, which was restored to near-normal levels in FSL-treated rats.

In conclusion, this study highlights the neuroprotective potential of FSL against ROT-induced dopaminergic neurodegeneration. The underlying mechanisms involve restoration of antioxidant defense, inhibition of inflammation, suppression of apoptosis, favorable regulation of autophagy, enhancement of neurogenesis, and reduction in α-synuclein. Collectively, these findings support the beneficial role of FSL in mitigating neurodegeneration. Given its commercial availability and use in dietary products, translating these effects to human trials is crucial for potential therapeutic or nutritional applications. Data from animal studies demonstrate negligible toxicity and neuroprotective potential in experimental models owing to the antioxidant and anti-inflammatory properties. Further regulatory toxicology and pharmacokinetic studies are required before prompting human usage. Although biochemical, molecular, and histological analyses provide strong evidence of neuroprotection, future studies should incorporate behavioral assessments to validate the functional significance of these findings. A key limitation of the current study is the lack of behavioral evaluations, such as motor coordination or rotarod performance tests, to assess the functional outcomes of FSL treatment. Nevertheless, FSL has been shown in other studies to improve cognitive behavior in neurodegenerative disease models.

## 4. Materials and Methods

### 4.1. Drugs and Chemicals

The highest purity ROT, FSL, dimethyl sulfoxide, myglyol, and paraformaldehyde were purchased from Sigma Aldrich in St. Louis, MO, USA. The glial fibrillary acidic protein (GFAP) and inducible nitric oxide synthase (iNOS) antibodies, as well as the RIPA buffer, were bought from Sigma Aldrich in St. Louis, MO, USA. The protease and phosphatase inhibitor cocktail was purchased from Thermo Fisher Scientific, Waltham, Massachusetts, USA. The polyclonal anti-tyrosine hydrolase (TH) antibody was obtained from Merck (Darmstadt, Germany). The antibodies, LC3, p62, mTOR, and phospho-mTOR were purchased from Cell Signaling Technology (Danvers, MA, USA). The monoclonal mouse anti-synuclein antibody and Iba-1 antibody were purchased from BD Biosciences (San Jose, CA, USA) and Wako Chemicals, Richmond, VA, USA, respectively. The apoptotic polyclonal markers Bax and Bcl-2 were procured from Abcam (Cambridge, MA, USA). The fluorescent secondary antibody, Alexa Fluor 488, used in the study was supplied by Thermo Fischer Scientific (Waltham, MA, USA). The secondary goat anti-rabbit biotinylated antibody was purchased from Jackson Immune Research Laboratory (West Grove, PA, USA). The commercially available kits were used to perform biochemical assays. All of the extra substances used in these experiments were of analytical grade and were bought from local vendors.

### 4.2. Animals and Study Design

Animal experiments were executed in compliance with the standards of the Animal Research Ethics Committee at United Arab Emirates University (Ethical Approval Number: 2023_2635). Male Wistar rats (6 weeks old, 180–220 g) were housed under constant conditions of light and darkness in cycles of 12 h each with relative standard humidity and temperature conditions. Rats were allowed to freely access food and water and acclimatized for one week before experiments. In each cage, 5 rats were grouped. In this study, rats were categorized into four groups with fifteen in each. Group 1 (CON) control rats received oral olive oil as a vehicle for 4 weeks. Group 2 (ROT) rats received ROT (2.5 mg/kg, i.p.) for 4 weeks [6]. Group 3 (FSL) rats received FSL (50 mg/kg p.o., twice daily) for 4 weeks [54]. Group 4 (FSL + ROT) rats were administered with FSL (50 mg/kg, p.o., twice daily), followed by i.p. injection of rotenone (2.5 mg/kg) with an interval of one hour for four weeks.

### 4.3. Tissue Collection

A dose of 40 mg/kg of pentobarbital sodium was used to anesthetize the rats at the end of the treatment in the adopted experimental protocol. Cardiac perfusion was performed using phosphate-buffered saline (0.01 M) to eliminate blood. The midbrain and striatum were excised from the brain and immediately frozen in liquid nitrogen. To perform the immunochemical analysis, the full brain was post-fixed in 4% paraformaldehyde.

### 4.4. Biochemical Analysis

For the biochemical estimation, extracted midbrain samples were homogenized in RIPA buffer with the addition of protease and phosphatase inhibitors acquired from Thermo Fisher Scientific (Rockford, IL, USA) using an electric homogenizer. The homogenate was further centrifuged at 14,000× *g* for 20 min at 4 °C, and the supernatant was used for further analysis.

### 4.5. Detection of the SOD, Catalase Activities, and Glutathione (GSH) Concentration

Following the manufacturer’s protocol, assay kits (Cayman Chemicals Co., Ann Arbor, MI, USA; Sigma-Aldrich, St. Louis, MO, USA) were used to determine the activity of SOD, catalase, and the concentration of GSH in the collected samples. A microplate reader was used to read the absorbance at 535 nm, and readings were expressed as U/mL, nmol/min/mL, and µM/mL for SOD, catalase, and GSH, respectively.

### 4.6. Detection of Malondialdehyde (MDA) Level

The level of MDA was estimated by a commercially available assay kit (Northwest Life Science, Vancouver, WA, USA), and readings were expressed as µM/mL.

### 4.7. Detection of Proinflammatory Cytokine Levels

The levels of tumor necrosis factor-alpha (TNF-α), interleukin-1β (IL-1β), and interleukin-6 (IL-6) were estimated following the enzyme-linked immunosorbent assay (ELISA) kit (BioSource International, Camarillo, CA, USA) and readings expressed as pg/mL.

### 4.8. Western Blotting

In a similar manner to the biochemical analysis tissue preparation, the striatal tissues isolated from the brains of different groups were homogenized using RIPA buffer (Millipore, Burlington, MA, USA) with protein and protease inhibitors (Merck Millipore, Burlington, MA, USA). Tissue homogenates were centrifuged at 14,000× *g* for 20 min at 4 °C to yield the supernatant used for later analysis. The obtained supernatant was dissolved in 4× Laemmli visualizing buffer (Bio Rad, Hercules, CA, USA) and mixed with 2-mercaptoethanol (Sigma Chemicals, St. Louis, MO, USA). The concentration of protein in samples was estimated using Pierce™ BCA protein assay kit (Thermo Fisher Scientific, Rockford, IL, USA). The sample protein was loaded in equal amounts for SDS gel electrophoresis. The blocking was performed using 5% milk in PBST after transferring to a PVDF membrane. The membrane was then incubated with different primary antibodies overnight on a constant agitator at 4 °C, followed by horseradish peroxidase-linked secondary antibody incubation for 1 h at room temperature. ECL reagents (Thermo Fisher Scientific, Rockford, IL, USA) were used for detecting the expressed signal. Primary antibodies used include anti-iNOS (1:2000), (Sigma Chemicals, St. Louis, MO, USA), anti-COX-2 (1:1000), anti-caspase-3 (1:1000), anti-cleaved caspase-3 (1:500), anti-caspase-9 (1:1000), anti-cleaved caspase-9, (1:500), anti-cytochrome-C (1:2000), anti-mTOR (1:1000), anti-phospho-mTOR (1:500), anti-P62 (1:1000), anti-phospho-IκB (1:500), anti-IκB (1:1000) (Cell Signaling Technology, Danvers, MA, USA), anti α-Syn (1:1000), anti-P38 (1:1000), anti-mitochondrial complex I (1:2000), anti-brain-derived neurotropic factor (BDNF) (1:1000), anti-P38 MAPK (1:2000), anti-phospho-P38 MAPK (1:1000), anti-Bcl-2 (1:500), anti-BCL-xL (1:1000), anti-phospho-NF-κB (1:500), anti-Bax (1:100), (Santa Cruz Biotechnology, Dallas, TA, USA), anti-TH (1:1000) antibody was (Merck, Darmstadt, Germany), anti-TNF-α (1: 1000), anti-IL-6 (1:1000), anti-IL-1β (1:1000), and anti-TLR4 (1:1000) (Abcam, MA, USA).

### 4.9. Immunofluorescence Staining for Glial Fibrillary Acidic Protein (GFAP) and Ionized Calcium-Binding Adapter Molecule 1 (Iba-1)

The brain tissues were sectioned from bregma 1.20 to −8.0 mm. The interaural position was 10.20 mm to 8.20. Serial sections were taken (25 µm), and sections stained were selected at intervals of 10 serial sections. 25 µm sections of the striatum were cut by microtome and then immersed in blocking solution containing 10% normal goat serum and 0.3% Triton-X 100 (Sigma Aldrich, St. Louis, MO, USA) in PBS for one hour. After the blocking step, sections were incubated with primary antibodies of polyclonal rabbit anti-GFAP (1:1000) (Abcam, Waltham, MA, USA) and anti-Iba-1 (1:1000) (Vako Chemicals, Richmond, VA, USA) overnight at 4 °C. The next day, the sections were rinsed and incubated in Alexa Fluor^®^ 488 anti-rabbit secondary antibody for one hour and then mounted with mounting medium Fluoroshield™ (Sigma-Aldrich, St. Louis, MO, USA). The slides were viewed under a fluorescent microscope, EVOS FL (Thermo Fisher Scientific, Waltham, MA, USA).

### 4.10. Detection of GFAP and Iba-1 Activation

The activation of glial and astrocytes was indicated by increased green fluorescent intensity shown under the microscope. Multiple sections of the same area were analyzed and quantified by ImageJ (version 1.54k) (NIH, Bethesda, MD, USA).

### 4.11. Immunostaining of Tyrosine Hydroxylase (TH)

Sections of striatum and substantia nigra were cut at a thickness of 25 µm by cryostat (Leica, Wetzlar, Germany). Sections were processed by blocking with 10% normal goat serum and 0.3% Triton-X 100 for one hour. Furthermore, the sections were incubated with a primary antibody, rabbit anti-TH (1:500) (Millipore, Burlington, MA, USA), overnight at 4 °C. Thereafter, the sections were washed and incubated with the secondary anti-rabbit antibody (1:1000) for one hour. The immunoreactivity was completed by incubation with avidin–biotin complex (Vector Laboratories Ltd., Burlingame, CA, USA) and 3,3-diaminobenzidine (DAB). The slides were finally viewed under the light microscope (Olympus, Hamburg, Germany).

### 4.12. Detection of the Immunoreactivity of TH Neurons in the Striatum and TH Fibers in the Substantia Nigra

The number of stained immunoreactive neurons and fibers in the striatum and substantia nigra was quantified by counting the stained nuclei of neuronal soma. Same-dimensional areas from different slides were assessed for their optical density by ImageJ software, eliminating the overlapping optical density of the cortex. The assessment of immunoreactivity was performed by an investigator unaware of the treatment and experimental groups.

### 4.13. Statistical Analysis

All data are presented as mean ± standard error of the mean (SEM). Statistical analyses were performed using IBM SPSS Statistics version 29.0.0.0 (241). Differences among groups were evaluated by one-way analysis of variance (ANOVA), followed by Duncan’s Multiple Range Test (DMRT) for post hoc comparisons. A *p*-value ≤ 0.05 was considered statistically significant. All experiments were conducted in replicates, and the number of animals per group is specified in the corresponding figure legends.

## Data Availability

The data and interpretations are included in the manuscript.

## References

[1] Dias V., Junn E., Mouradian M.M. (2013). The role of oxidative stress in Parkinson’s disease. J. Park. Dis..

[2] Hald A., Lotharius J. (2005). Oxidative stress and inflammation in Parkinson’s disease: Is there a causal link?. Exp. Neurol..

[3] Lecours C., Bordeleau M., Cantin L., Parent M., Paolo T.D., Tremblay M. (2018). Microglial Implication in Parkinson’s Disease: Loss of Beneficial Physiological Roles or Gain of Inflammatory Functions?. Front. Cell Neurosci..

[4] Badanjak K., Fixemer S., Smajić S., Skupin A., Grünewald A. (2021). The Contribution of Microglia to Neuroinflammation in Parkinson’s Disease. Int. J. Mol. Sci..

[5] Iqubal A., Syed M.A., Najmi A.K., Azam F., Barreto G.E., Iqubal M.K., Ali J., Haque S.E. (2020). Nano-engineered nerolidol loaded lipid carrier delivery system attenuates cyclophosphamide neurotoxicity—Probable role of NLRP3 inflammasome and caspase-1. Exp. Neurol..

[6] Javed H., Azimullah S., Haque M.E., Ojha S.K. (2016). Cannabinoid Type 2 (CB2) Receptors Activation Protects against Oxidative Stress and Neuroinflammation Associated Dopaminergic Neurodegeneration in Rotenone Model of Parkinson’s Disease. Front. Neurosci..

[7] Mayne K., White J.A., McMurran C.E., Rivera F.J., de la Fuente A.G. (2020). Aging and Neurodegenerative Disease: Is the Adaptive Immune System a Friend or Foe?. Front. Aging Neurosci..

[8] Sánchez-Pernaute R., Ferree A., Cooper O., Yu M., Brownell A.L., Isacson O. (2004). Selective COX-2 inhibition prevents progressive dopamine neuron degeneration in a rat model of Parkinson’s disease. J. Neuroinflamm..

[9] Devi L., Raghavendran V., Prabhu B.M., Avadhani N.G., Anandatheerthavarada H.K. (2008). Mitochondrial import and accumulation of alpha-synuclein impair complex I in human dopaminergic neuronal cultures and Parkinson disease brain. J. Biol. Chem..

[10] Heidari A., Yazdanpanah N., Rezaei N. (2022). The role of Toll-like receptors and neuroinflammation in Parkinson’s disease. J. Neuroinflamm..

[11] Wong M. (2013). Mammalian target of rapamycin (mTOR) pathways in neurological diseases. Biomed. J..

[12] Azimullah S., Meeran M.F.N., Ayoob K., Arunachalam S., Ojha S., Beiram R. (2023). Tannic Acid Mitigates Rotenone-Induced Dopaminergic Neurodegeneration by Inhibiting Inflammation, Oxidative Stress, Apoptosis, and Glutamate Toxicity in Rats. Int. J. Mol. Sci..

[13] Teeter M.E., Baginsky M.L., Hatefi Y. (1969). Ectopic inhibition of the complexes of the electron transport system by rotenone, piericidin A, demerol and antimycin A. Biochim. Biophys. Acta.

[14] Johnson M.E., Bobrovskaya L. (2015). An update on the rotenone models of Parkinson’s disease: Their ability to reproduce the features of clinical disease and model gene-environment interactions. Neurotoxicology.

[15] Wen S., Aki T., Unuma K., Uemura K. (2020). Chemically Induced Models of Parkinson’s Disease: History and Perspectives for the Involvement of Ferroptosis. Front. Cell. Neurosci..

[16] Santhanasabapathy R., Sudhandiran G. (2015). Farnesol attenuates lipopolysaccharide-induced neurodegeneration in Swiss albino mice by regulating intrinsic apoptotic cascade. Brain Res..

[17] Santhanasabapathy R., Vasudevan S., Anupriya K., Pabitha R., Sudhandiran G. (2015). Farnesol quells oxidative stress, reactive gliosis and inflammation during acrylamide-induced neurotoxicity: Behavioral and biochemical evidence. Neuroscience.

[18] Li Y., Xie Z., Luo X., Wang X., Wang Y., Guo M., Zhou Z., Sun R., Hua D., Luo A. (2023). Farnesol Exerts Protective Effects against Chronic Sleep Deprivation-Induced Cognitive Impairment via Activation SIRT1/Nrf2 Pathway in the Hippocampi of Adult Mice. Mol. Nutr. Food Res..

[19] Javed H., Azimullah S., Abul Khair S.B., Ojha S., Haque M.E. (2016). Neuroprotective effect of nerolidol against neuroinflammation and oxidative stress induced by rotenone. BMC Neurosci..

[20] Cheong S.L., Federico S., Spalluto G., Klotz K.-N., Pastorin G. (2019). The current status of pharmacotherapy for the treatment of Parkinson’s disease: Transition from single-target to multitarget therapy. Drug Discov. Today.

[21] Ntetsika T., Papathoma P.-E., Markaki I. (2021). Novel targeted therapies for Parkinson’s disease. Mol. Med..

[22] Feraco P., Gagliardo C., La Tona G., Bruno E., D’angelo C., Marrale M., Del Poggio A., Malaguti M.C., Geraci L., Baschi R. (2021). Imaging of Substantia Nigra in Parkinson’s Disease: A Narrative Review. Brain Sci..

[23] Gao F., Chen D., Hu Q., Wang G. (2013). Rotenone directly induces BV2 cell activation via the p38 MAPK pathway. PLoS ONE.

[24] Jiang X., Feng X., Huang H., Liu L., Qiao L., Zhang B., Yu W. (2017). The effects of rotenone-induced toxicity via the NF-κB–iNOS pathway in rat liver. Toxicol. Mech. Methods.

[25] Beraud D., Twomey M., Bloom B., Mittereder A., Neitzke K., Ton V., Chasovskikh S., Mhyre T., Maguire-Zeiss K. (2011). α-Synuclein Alters Toll-Like Receptor Expression. Front. Neurosci..

[26] Conte C., Ingrassia A., Breve J., Bol J.J., Timmermans-Huisman E., van Dam A.-M., Beccari T., van de Berg W.D.J. (2023). Toll-like Receptor 4 Is Upregulated in Parkinson’s Disease Patients and Co-Localizes with pSer129αSyn: A Possible Link with the Pathology. Cells.

[27] Ishola I.O., Awogbindin I.O., Olubodun-Obadun T.G., Oluwafemi O.A., Onuelu J.E., Adeyemi O.O. (2022). Morin ameliorates rotenone-induced Parkinson disease in mice through antioxidation and anti-neuroinflammation: Gut-brain axis involvement. Brain Res..

[28] Rempe R.G., Hartz A.M.S., Bauer B. (2016). Matrix metalloproteinases in the brain and blood-brain barrier: Versatile breakers and makers. J. Cereb. Blood Flow Metab..

[29] Corrêa S.A.L., Eales K.L. (2012). The Role of p38 MAPK and Its Substrates in Neuronal Plasticity and Neurodegenerative Disease. J. Signal Transduct..

[30] Fato R., Bergamini C., Bortolus M., Maniero A.L., Leoni S., Ohnishi T., Lenaz G. (2009). Differential effects of mitochondrial Complex I inhibitors on production of reactive oxygen species. Biochim. Biophys. Acta.

[31] Li N., Ragheb K., Lawler G., Sturgis J., Rajwa B., Melendez J.A., Robinson J.P. (2003). Mitochondrial complex I inhibitor rotenone induces apoptosis through enhancing mitochondrial reactive oxygen species production. J. Biol. Chem..

[32] Arab H.H., Safar M.M., Shahin N.N. (2021). Targeting ROS-Dependent AKT/GSK-3β/NF-κB and DJ-1/Nrf2 Pathways by Dapagliflozin Attenuates Neuronal Injury and Motor Dysfunction in Rotenone-Induced Parkinson’s Disease Rat Model. ACS Chem. Neurosci..

[33] Ighodaro O.M., Akinloye O.A. (2018). First line defence antioxidants-superoxide dismutase (SOD), catalase (CAT) and glutathione peroxidase (GPX): Their fundamental role in the entire antioxidant defence grid. Alex. J. Med..

[34] Trist B.G., Hare D.J., Double K.L. (2019). Oxidative stress in the aging substantia nigra and the etiology of Parkinson’s disease. Aging Cell.

[35] Abukhalil M.H., Hussein O.E., Bin-Jumah M., Saghir S.A.M., Germoush M.O., Elgebaly H.A., Mosa N.M., Hamad I., Qarmush M.M., Hassanein E.M. (2020). Farnesol attenuates oxidative stress and liver injury and modulates fatty acid synthase and acetyl-CoA carboxylase in high cholesterol-fed rats. Environ. Sci. Pollut. Res. Int..

[36] Khan R., Sultana S. (2011). Farnesol attenuates 1,2-dimethylhydrazine induced oxidative stress, inflammation and apoptotic responses in the colon of Wistar rats. Chem. Biol. Interact..

[37] Redza-Dutordoir M., Averill-Bates D.A. (2016). Activation of apoptosis signalling pathways by reactive oxygen species. Biochim. Biophys. Acta (BBA).-Mol. Cell Res..

[38] Newhouse K., Hsuan S.L., Chang S.H., Cai B., Wang Y., Xia Z. (2004). Rotenone-induced apoptosis is mediated by p38 and JNK MAP kinases in human dopaminergic SH-SY5Y cells. Toxicol. Sci..

[39] Lizama B.N., Chu C.T. (2021). Neuronal autophagy and mitophagy in Parkinson’s disease. Mol. Asp. Med..

[40] Zhu Z., Yang C., Iyaswamy A., Krishnamoorthi S., Sreenivasmurthy S.G., Liu J., Wang Z., Tong B.C., Song J., Lu J. (2019). Balancing mTOR Signaling and Autophagy in the Treatment of Parkinson’s Disease. Int. J. Mol. Sci..

[41] Jayaraj R.L., Beiram R., Azimullah S., MF N.M., Ojha S.K., Adem A., Jalal F.Y. (2021). Noscapine Prevents Rotenone-Induced Neurotoxicity: Involvement of Oxidative Stress, Neuroinflammation and Autophagy Pathways. Molecules.

[42] Xilouri M., Vogiatzi T., Vekrellis K., Stefanis L. (2008). alpha-synuclein degradation by autophagic pathways: A potential key to Parkinson’s disease pathogenesis. Autophagy.

[43] Liu J., Liu W., Yang H. (2019). Balancing Apoptosis and Autophagy for Parkinson’s Disease Therapy: Targeting BCL-2. ACS Chem. Neurosci..

[44] Lan A.P., Chen J., Zhao Y., Chai Z., Hu Y. (2017). mTOR Signaling in Parkinson’s Disease. Neuromol. Med..

[45] Zhou Q., Liu C., Liu W., Zhang H., Zhang R., Liu J., Zhang J., Xu C., Liu L., Huang S. (2015). Rotenone induction of hydrogen peroxide inhibits mTOR-mediated S6K1 and 4E-BP1/eIF4E pathways, leading to neuronal apoptosis. Toxicol. Sci..

[46] Selvaraj S., Sun Y., Watt J.A., Wang S., Lei S., Birnbaumer L., Singh B.B. (2012). Neurotoxin-induced ER stress in mouse dopaminergic neurons involves downregulation of TRPC1 and inhibition of AKT/mTOR signaling. J. Clin. Investig..

[47] Motawi T.K., Al-Kady R.H., Abdelraouf S.M., Senousy M.A. (2022). Empagliflozin alleviates endoplasmic reticulum stress and augments autophagy in rotenone-induced Parkinson’s disease in rats: Targeting the GRP78/PERK/eIF2α/CHOP pathway and miR-211-5p. Chem.-Biol. Interact..

[48] Liu W.J., Ye L., Huang W.F., Guo L.J., Xu Z.G., Wu H.L., Yang C., Liu H.F. (2016). p62 links the autophagy pathway and the ubiqutin–proteasome system upon ubiquitinated protein degradation. Cell. Mol. Biol. Lett..

[49] Calabresi P., Mechelli A., Natale G., Volpicelli-Daley L., Di Lazzaro G., Ghiglieri V. (2023). Alpha-synuclein in Parkinson’s disease and other synucleinopathies: From overt neurodegeneration back to early synaptic dysfunction. Cell Death Dis..

[50] Rocha S.M., Bantle C.M., Aboellail T., Chatterjee D., Smeyne R.J., Tjalkens R.B. (2022). Rotenone induces regionally distinct α-synuclein protein aggregation and activation of glia prior to loss of dopaminergic neurons in C57Bl/6 mice. Neurobiol. Dis..

[51] Palasz E., Wysocka A., Gasiorowska A., Chalimoniuk M., Niewiadomski W., Niewiadomska G. (2020). BDNF as a Promising Therapeutic Agent in Parkinson’s Disease. Int. J. Mol. Sci..

[52] Johnson M.E., Lim Y., Senthilkumaran M., Zhou X.F., Bobrovskaya L. (2015). Investigation of tyrosine hydroxylase and BDNF in a low-dose rotenone model of Parkinson’s disease. J. Chem. Neuroanat..

[53] Johnson M.E., Zhou X.F., Bobrovskaya L. (2019). The effects of rotenone on TH, BDNF and BDNF-related proteins in the brain and periphery: Relevance to early Parkinson’s disease. J. Chem. Neuroanat..

[54] Qamar W., Sultana S. (2008). Farnesol ameliorates massive inflammation, oxidative stress and lung injury induced by intratracheal instillation of cigarette smoke extract in rats: An initial step in lung chemoprevention. Chem. Biol. Interact..

